# CircRNA microarray profiling identifies a novel circulating biomarker for detection of gastric cancer

**DOI:** 10.1186/s12943-018-0888-8

**Published:** 2018-09-20

**Authors:** Weiwei Tang, Kai Fu, Handong Sun, Dawei Rong, Hanjin Wang, Hongyong Cao

**Affiliations:** Department of general surgery, Nanjing First Hospital, Nanjing Medical University, Nanjing, Jiangsu China

**Keywords:** circRNAs, Microarray, Biomarker, Exosomes, Plasma

## Abstract

CircRNA expression profiles for gastric cancer (GC) were screened using plasma samples from 10 GC patients with different TNM stages and 5 healthy individuals as controls. Results showed lower expression of circ-KIAA1244 in GC tissues, plasmas, and cells compare to normal controls. Further clinical data analysis demonstrated that a decreased expression of circ-KIAA1244 in plasmas was negatively correlated with TNM stage and lymphatic metastasis, and a shorter overall survival time of GC patients. Moreover, we found that circ-KIAA1244 could be detected in GC plasma exosomes and showed no obvious significance compared to the expression level in the corresponding plasmas. This study revealed a GC-tissues-derived circ-KIAA1244 could serve a novel circulating biomarker for detection of GC.

**Electronic supplementary material:**

The online version of this article (10.1186/s12943-018-0888-8) contains supplementary material, which is available to authorized users.

## Main text

Circular RNAs (circRNAs) are a large new class of endogenous noncoding RNAs that form covalently closed continuous loops without 5′caps or 3′ tails [1]. Recently, a plethora of studies have reported associations between circRNAs and human diseases, especially carcinomas [2]. Exosomes are small membrane vesicles that can be isolated from serum, pleural effusions, urine, and ascites fluids of cancer patients [3]. Li Y. et al. first reported the presence of abundant circRNAs in exosomes by using RNA-seq analyses of ribosomal RNA-depleted total RNA from liver cancer cells and cell-derived exosomes [4]. In the present study, circRNA expression profiles were screened in clinical samples of gastric cancer (GC) patients with different TNM stages. Gene ontology (GO), Kyoto Encyclopedia of Genes and Genomes (KEGG) as well as disease pathways were further analyzed. Finally, candidate tumor-related circRNA was quantified in GC tissues, plasmas, plasma exosomes, and cell lines, respectively.

## CircRNA microarray

CapitalBio Technology Human CircRNA Array v2 was designed with four identical arrays per slide, with each array containing probes interrogating about 170,340 human circRNAs. Those circRNA target sequences were all from Circbase,Deepbase and Rybak-Wolf 2015.

## Patients and samples

Peripheral blood (5 ml) of 62 GC patients were obtained from before the operation during 2014–2017 and then the plasmas were isolated. Fresh control plasma samples were collected from 25 healthy people. A total of 28 pairs of GC tissues and corresponding adjacent non-tumor tissues were obtained from GC patients who underwent surgery at Nanjing First Hospital.

## RNA isolation, reverse transcription and quantitative real-time polymerase chain reaction

Total RNA from paired tissues or cells were extracted by using TRIzol reagent (Thermo Fisher Scientific, Waltham, MA, USA) and total RNA in plasma was extracted by TIANamp Virus RNA Kit following the manufacturer’s instructions. Quantitative real-time PCR was performed using SYBR Green and detected using the Applied Biosystems Real-Time PCR System. The comparative Ct method was used to compare each condition with the control reactions, and the values are expressed as-△Ct. Circ-KIAA1244 expression level was determined by qRT-PCR using the following primer pair: 5′- CAGTTACGACAGAGGCAGGA-3′ (Forward, or F) and 5′- CAGCAGGCATTTCTCCTTTATG-3′ (reverse, R). Glyceraldehyde 3-phosphate dehydrogenase (GAPDH) was used as an internal control.

## Plasma exosome extraction

All the steps were performed following the instructions of Hieff™ Quick exosome isolation kit (for Serum/Plasma, Shanghai, 41202ES30). We transferred 1 mL of blood sample to a centrifuge tube, centrifuge at 3000×g for 10 min at 4 °C, discarded the pellet, and transferred the supernatant to a new centrifuge tube. Then we transferred the supernatant to a new centrifuge tube. To the pretreated sample, 4 volumes of 1 x PBS were added and mixed well.

## RNA fluorescence in situ hybridization (RNA-FISH)

The RNA FISH probe of circ-KIAA1244 was synthesized by Ribo Bio Technology Co. Ltd. (Guangzhou, China). Cells were hybridized with RNA of a mixture of 20 μM Ca3 labeled FISH probe of circ-KIAA1244 at 37 °C overnight and were stained with 4,6-diamidino-2-phenylindole (DAPI) for 10 min. All images were acquired by using confocal microscope.

## RNA immunoprecipitation (RIP)

All the steps were performed following the instructions of Magna RIP RNA-Binding Protein Immunoprecipitation Kit (Millipore, Bedford, MA). The 100 ml cell lysates were incubated with human anti-AGO2 antibody (Abcam, USA) or negative control mouse IgG(Millipore, USA) at 4 °C overnight. The immunoprecipitated RNAs were extracted using the RNeasy MinElute Cleanup Kit (Qiagen, China) and reverse transcribed using the Goldenstar™ RT6 cDNA Synthesis Kit (TSINGKE, Beijing, China).

## Statistical analysis

Continuous data were analyzed using an independent t-test between the two groups. Receiver-operating characteristic (ROC) curves and the area under the ROC curve (AUC) were used to assess the diagnostic performance of circ-KIAA1244. The relationship between circ-KIAA1244 expression and overall survival time was evaluated by Kaplan-Meier plots and log-rank tests. Statistical analysis was performed with SPSS (Version 22.0, IBM, USA) and presented graphically in GraphPad Prism 5.0. A *P*-value of 0.05 was considered to be statistically significant.

## Findings

### CircRNA expression profiles in GC progression and bioinformatics prediction analysis

**Fig. 1:**
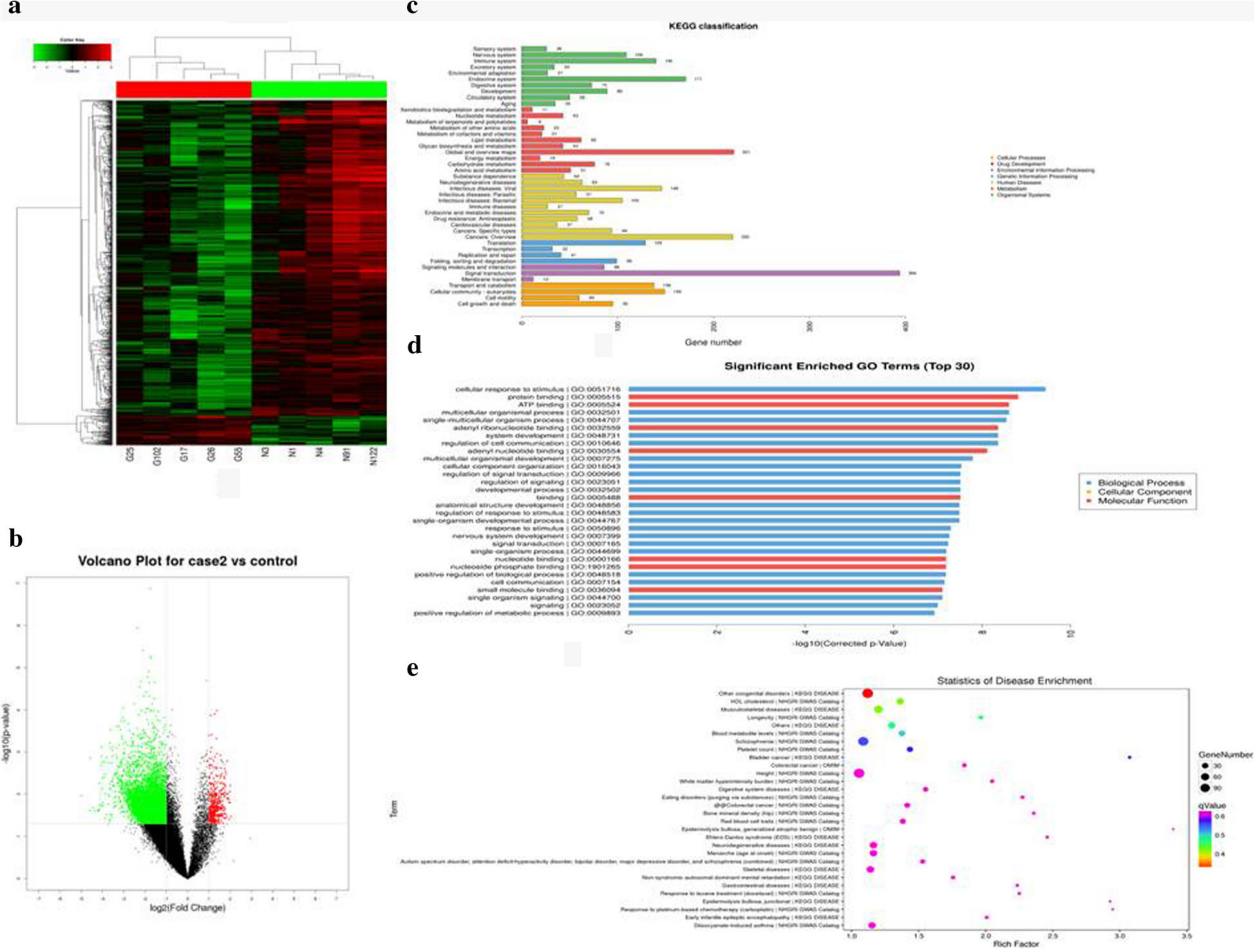
Profiling of circular RNAs in the plasmas from GC patients with early TNM stages and normal controls. (**a**) Heat map shows the up-regulated and down-regulated circRNAs in case1 vs control group. (G for GC, and N for normal individuals’ plasma). Each column represents the expression profile of a tissue sample, and each row corresponds to a circRNA. High expression level is indicated by “red” and lower levels by “green”. (**b**) Volcano plot shows the up-regulated and down-regulated circRNAs in case1 vs control group. Higher expression levels are indicated by “red”, lower expression levels are indicated by “green”, and no significant difference is indicated by “black”. (**c**) KEGG analysis of circRNAs in case1 vs control group. (**d**) GO analysis of circRNAs in case1 vs control group. (**e**) Disease pathway analysis of circRNAs in case1 vs control group

High-throughput human circRNA microarray was conducted using plasma samples from 10 GC patients, including 5 patients with T1-3N0M0 (case1) and the other 5 patients with T3 N1-3 M0 (case2), and 5 normal individuals (control) to assess circRNA expression profiles in GC progression. The data were presented in the format of Heat maps (Fig. 1a) and Volcano plots (Fig. 1b) in case1 vs control group. GO, KEGG and disease pathway analysis suggest that these differentially expressed circRNAs are relevant to several vital physiological processes, cellular components, molecular functions, and critical signaling pathways (Fig. 1c-e). Of course, the other two groups, including case2 vs control (Additional file 1: Figure S1a-e) and case2 vs case1 (Additional file 2: Figure S2a-e) were showed in the same way. Given that circRNAs are able to bind to miRNA [5], we investigated the relation of circRNA and possible binding miRNAs in different groups, which was presented in Additional file 3: Figure S3a-c.

### The biological structure of circ-KIAA1244

**Fig. 2:**
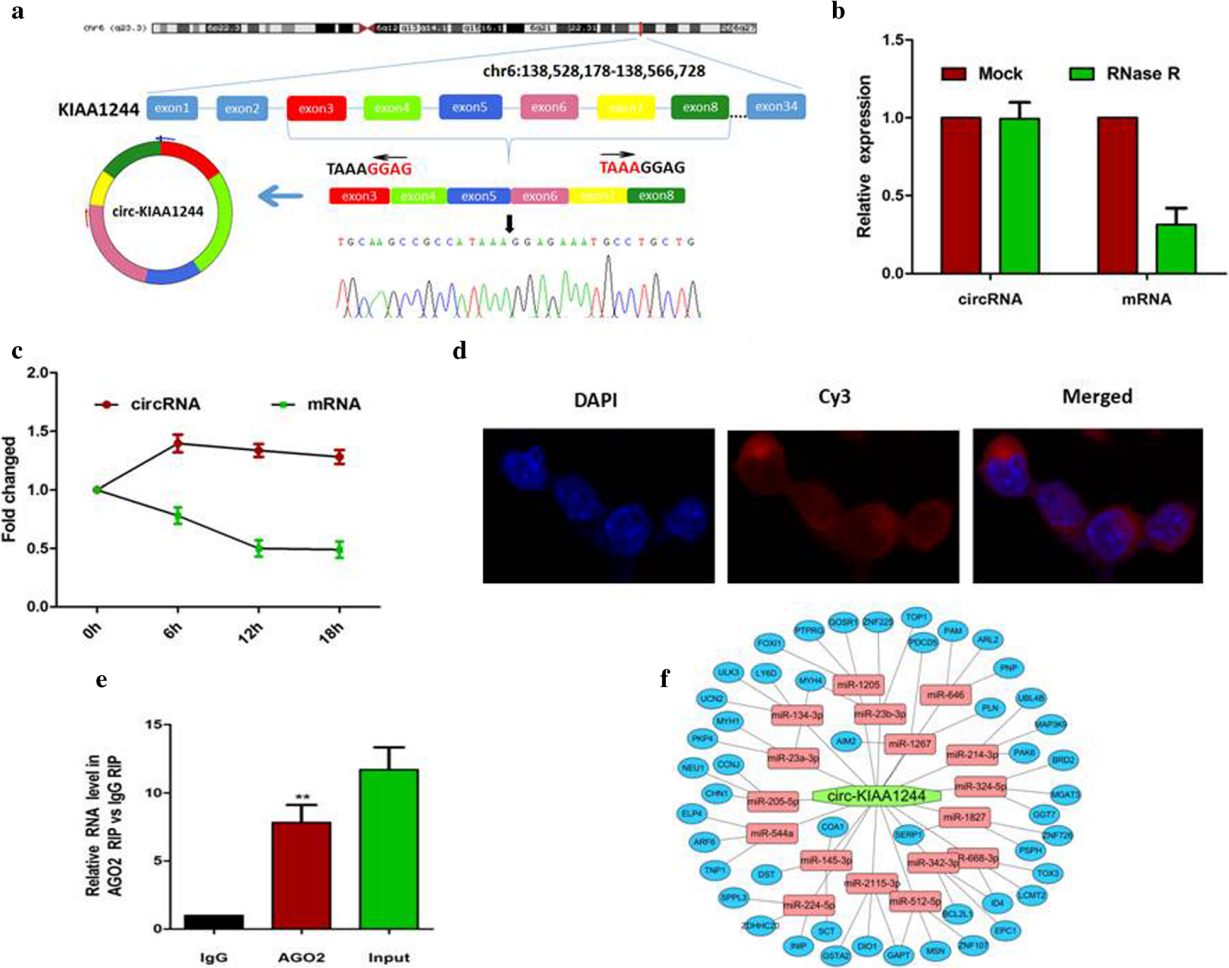
The biological structure of circ-KIAA1244. (**a**) Schematics showed that circ-KIAA1244 is derived from KIAA1244 exons 3–8. The amplification products were inserted into a T-vector for Sanger sequencing to determine their full-length. (**b**) qRT-PCR for the abundance of circ-KIAA1244 and KIAA1244 mRNA in GC cells treated with RNase R. (**c**) qRT-PCR for the abundance of circ-KIAA1244and KIAA1244 mRNA in GC cells treated with actinomycin D. (**d**) circ-KIAA1244 was mainly localized in the cytoplasm. (**e**) circ-KIAA1244 was enriched in Ago2-containing immunoprecipitates compared with control immunoglobulin G (IgG) immunoprecipitates. (**f**) The possible binding miRNAs and mRNAs of circ-KIAA1244. **P<0.01

We selected a total of 8 circRNAs based on the multiple fold difference between the expression of GC plasmas and normal controls and then verified the findings in a small sample of plasmas by using qRT-PCR. Results showed that circ-KIAA1244 (hsa_circ_0130810 is in gene symbol KIAA1244 and thus, we named it as circ-KIAA1244) has a significantly lower expression in GC plasmas compared to normal controls (Additional file 4: Figure S4). Circ-KIAA1244 is derived from the exons 3–8 of the gene KIAA1244 and the amplification products were inserted into a T-vector for Sanger sequencing to determine its full-length (Fig. 2a). Resistance to digestion with RNase R (Fig. 2b) or actinomycin D (Fig. 2c) further confirmed this RNA species was circular in form. FISH experiment demonstrated that the circular form of KIAA1244 was mainly localized in the cytoplasm (Fig. 2d). RNA immunoprecipitation was performed on GC cells and the result showed that circ-KIAA1244 was enriched in Ago2-containing immunoprecipitates compared with control immunoglobulin G (IgG) immunoprecipitates (Fig. 2e). Based on the predictions of circbank (http://www.circbank.cn/), we found that circ-KIAA1244 can bind to 17 miRNAs, and further we predicted three target mRNAs for each miRNA in the Targetscan database (http://www.targetscan.org),which was shown in Fig. 2f.

### The expressions of circ-KIAA1244 in GC samples and clinical information analysis

**Fig. 3:**
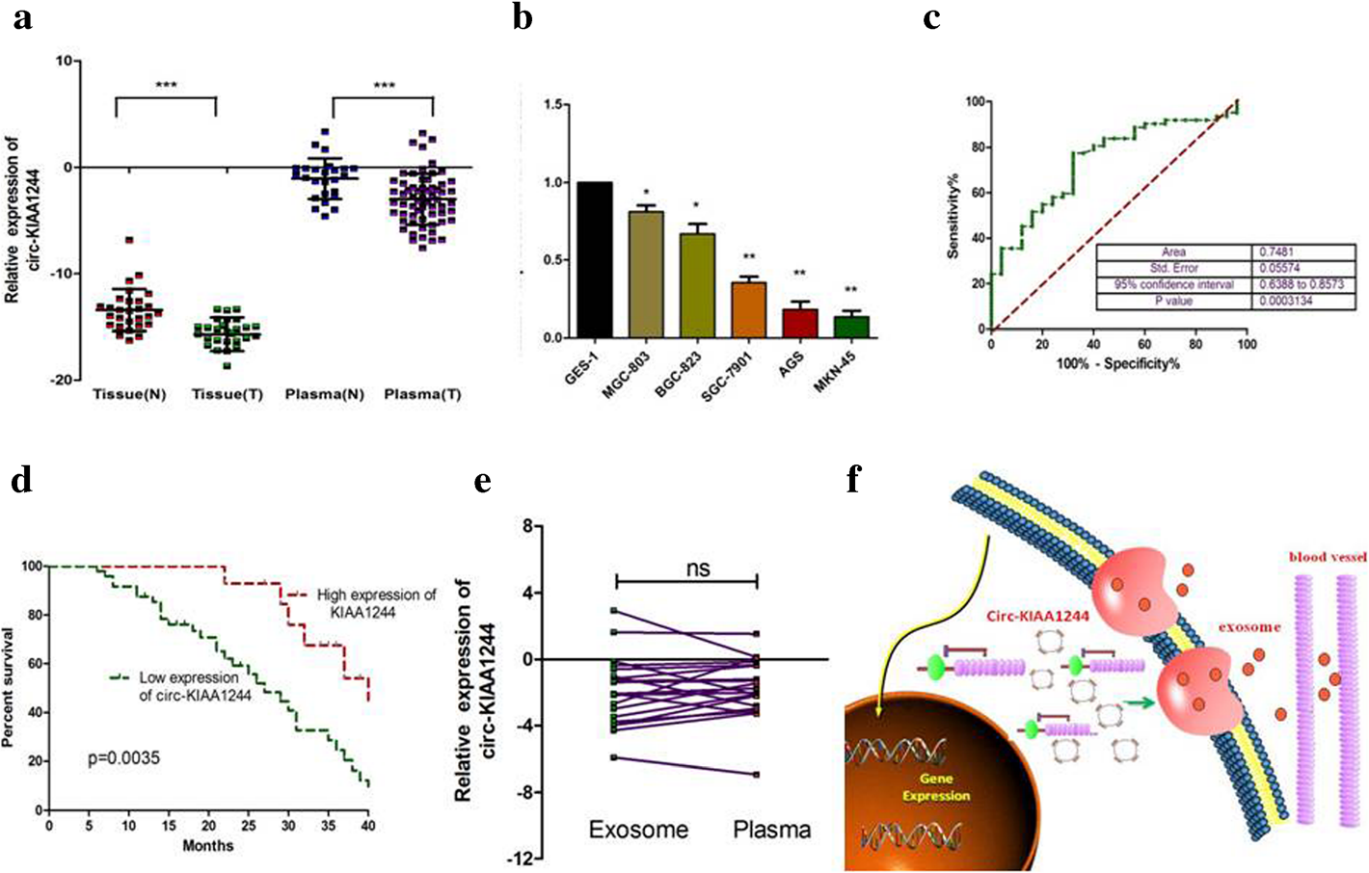
The expressions of circ-KIAA1244 GC samples and clinical information analysis. (**a**) circ-KIAA1244 expression was down-regulated in GC tissues and plasmas compared to normal controls. The correlation of their -ΔCt value was determined. N indicates normal and T indicates tumor. (**b**) The expression of circ-KIAA1244 in GC cell lines was lower than that in GES-1 significantly. (**c**) The area under the ROC curve (AUC) in distinguishing GC plasmas and normal ones was 0.7481. (**d**) Kaplan-Meier overall survival curve revealed patients with lower circ-KIAA1244 expression showed a reduced survival time. (**e**) There was no significant difference between the expression of circ-KIAA1244 in plasmas and corresponding plasma exosomes. (**f**) Theoretical model diagram of circ-KIAA1244 in GC: we proposed that GC-tissues-derived circ-KIAA1244 might be transmitted to plasmas via exosomes delivery and could serve as a biomarker in GC.*P<0.05, **P<0.01, ***P<0.001

Given that the tremendous diagnostic and therapeutic role of circRNAs in GC, we explored the clinical value of circ-KIAA1244 by detecting its expression in GC samples. Results indicated circ-KIAA1244 had significantly lower expression in GC plasma, tissues and cells compared to normal controls (Fig. 3a-b). Clinicopathological features showed that down-expression of circ-KIAA1244 was negatively associated with TNM stage and lymphatic metastasis (Additional file 5: Table S1). Furthermore, the area under the ROC curve (AUC) of circ-KIAA1244 in distinguishing GC plasmas and normal ones was 0.7481 (Fig. 2c) and the cut-off value was − 1.443 with the sensitivity of 77.42% and specificity of 68.00%. Kaplan-Meier overall survival curve revealed that patients with lower circ-KIAA1244 expression showed a reduced survival time (Fig. 3d). Univariate and multivariate analysis indicated that relative circ-KIAA1244 expression could serve as an independent prognostic indicator for the overall survival rate of patients with GC (Additional file 5: Table S2). According to the literature, plasma circRNAs can be encapsulated in exosomes [6], and we suspect that circ-KIAA1244 is present in plasma in the same way. Therefore, we divided the plasma from 21 GC patients into two parts, one directly measured the level of circ-KIAA1244 in plasmas, and the other detected the level of circ-KIAA1244 in the exosomes. The results showed that there was no significant difference between the two groups (Fig. 3e). Based on these experiments, we proposed that most of the circ-KIAA1244 is encapsulated in the exosomes, which can be decomposed by the large amount of RNase present in plasmas.

## Conclusion

Our research revealed a novel GC-tissues-derived circ-KIAA1244, which could serve a novel circulating biomarker for detection of GC (Fig. 3f).

## Additional file

Additional file 1: **Figure S1.** Profiling of circular RNAs in the plasmas from GC patients with advanced TNM stages and normal controls. (a) Heat map shows the up-regulated and down-regulated circRNAs in case2 vs control group. (G for GC, and N for normal individuals’ plasma). Each column represents the expression profile of a tissue sample, and each row corresponds to a circRNA. Higher expression levels are indicated by “red” and lower expression levels are indicated by “green”. (b) Volcano plot shows the up-regulated and down-regulated circRNAs in case2 vs control group. Higher expression levels are indicated by “red”, lower expression levels are indicated by “green”, and no significant difference is indicated by “black”. (c) KEGG analysis of circRNAs in case2 vs control group. (d) GO analysis of circRNAs in case2 vs control group. (e) disease pathway analysis of circRNAs in case2 vs control group. (JPG 69 kb)

Additional file 2: **Figure S2.** Profiling of circular RNAs in the plasmas from GC patients. (a) Heat map shows the up-regulated and down-regulated circRNAs in case2 vs case1 group. (G for GC, and N for normal individuals’ plasma). Each column represents the expression profile of a tissue sample, and each row corresponds to a circRNA. Higher expression levels are indicated by “red” and lower expression levels are indicated by “green”. (b) Volcano plot shows the up-regulated and down-regulated circRNAs in case2 vs control group. Higher expression levels are indicated by “red”, lower expression levels are indicated by “green”, and no significant difference is indicated by “black”. (c) KEGG analysis of circRNAs in case2 vs case1 group. (d) GO analysis of circRNAs in case2 vs case1group. (e) disease pathway analysis of circRNAs in case2 vs case1 group. (JPG 62 kb)

Additional file 3: **Figure S3.** The possible binding miRNAs of circRNAs. (a) The relationship between circRNA and possible binding miRNAs in case2 vs control group. (b) The relation of circRNA and possible binding miRNAs in case2 vs control group. (c) The relationship between circRNA and possible binding miRNAs in case2 vs case1 group. (JPG 58 kb)

Additional file 4: **Figure S4.** A total of 8 circRNAs based on the multiple fold difference between the expression of GC plasmas and normal controls were verified that in a small sample of plasmas by using qRT-PCR. *P<0.05, **P<0.01,***P<0.001. (JPG 31 kb)

Additional file 5: **Table S1.** The association of circ-KIAA1244 expression (-ΔCt) in plasma with baseline demographics of patients with GC. **Table S2.** Univariate and multivariate analysis for overall survival. (DOCX 24 kb)

## Abbreviations

circRNAs: Circular RNAs
GC: Gastric cancer
GO: Gene Oncology
IgG: Immunoglobulin G
KEGG: Kyoto Encyclopedia of Genes and Genomes
RIP: RNA immunoprecipitaion
RNA-FISH: RNA fluorescence in situ hybridization
ROC: Receiver-operating characteristic
