# Supplementary figures and images for: CircRNA microarray profiling identifies a novel circulating biomarker for detection of gastric cancer
Source: Mol Cancer. 2018 Sep 20;17:137. doi: 10.1186/s12943-018-0888-8 (PMC6147053; doi:10.1186/s12943-018-0888-8)

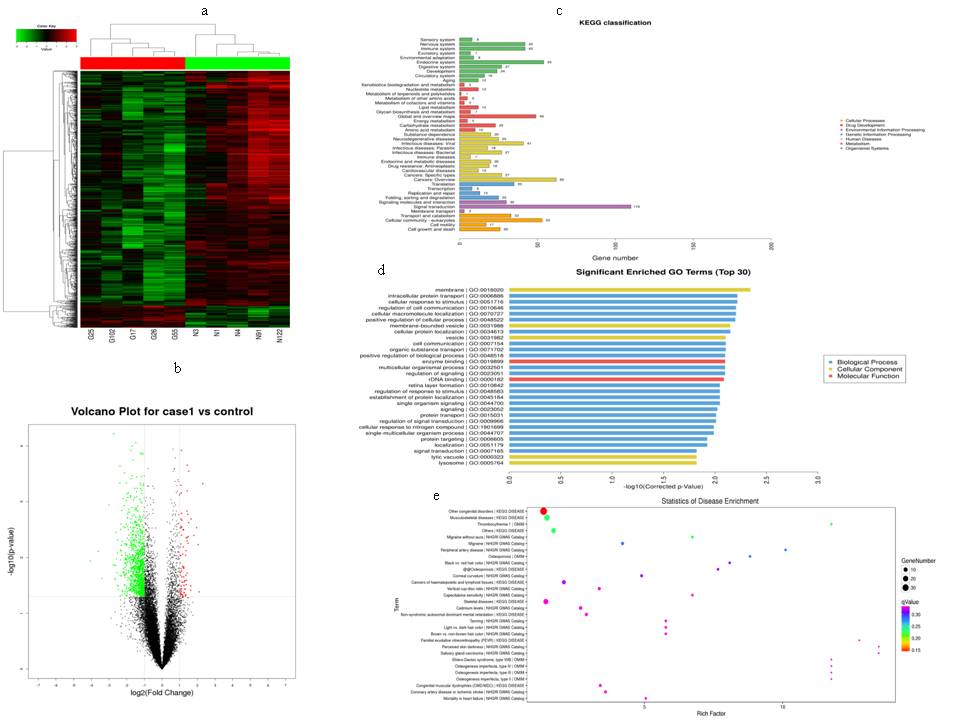

Supplement: Supplementary file 1 — Figure S1. Profiling of circular RNAs in the plasmas from GC patients with advanced TNM stages and normal controls. (a) Heat map shows the up-regulated and down-regulated circRNAs in case2 vs control group. (G for GC, and N for normal individuals’ plasma). Each column represents the expression profile of a tissue sample, and each row corresponds to a circRNA. Higher expression levels are indicated by “red” and lower expression levels are indicated by “green”. (b) Volcano plot shows the up-regulated and down-regulated circRNAs in case2 vs control group. Higher expression levels are indicated by “red”, lower expression levels are indicated by “green”, and no significant difference is indicated by “black”. (c) KEGG analysis of circRNAs in case2 vs control group. (d) GO analysis of circRNAs in case2 vs control group. (e) disease pathway analysis of circRNAs in case2 vs control group. (JPG 69 kb) [file 12943_2018_888_MOESM1_ESM.jpg]

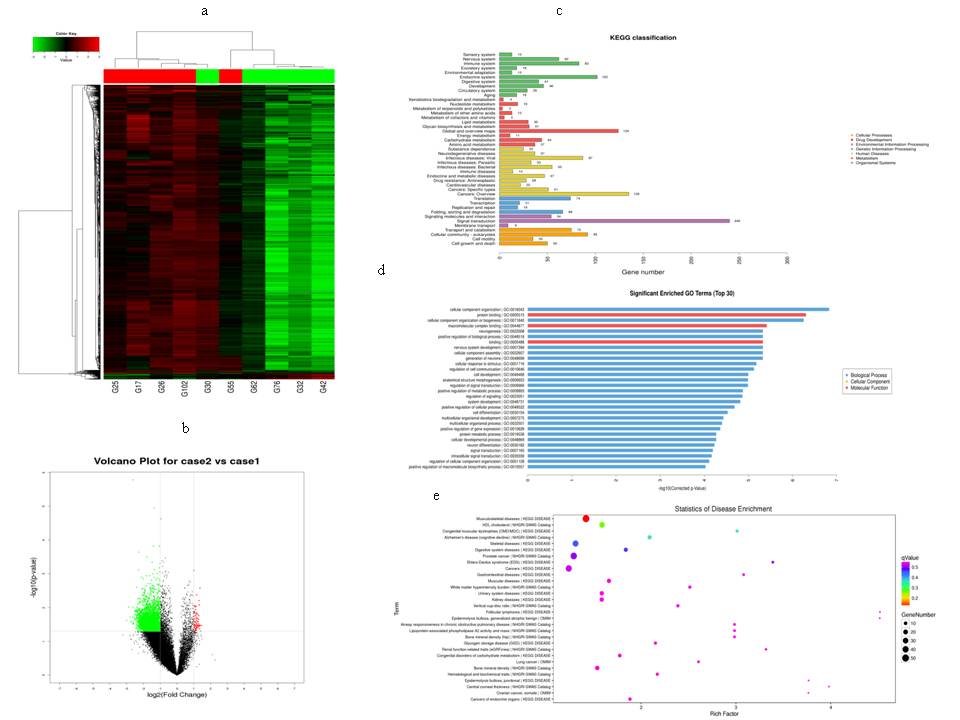

Supplement: Supplementary file 2 — Figure S2. Profiling of circular RNAs in the plasmas from GC patients. (a) Heat map shows the up-regulated and down-regulated circRNAs in case2 vs case1 group. (G for GC, and N for normal individuals’ plasma). Each column represents the expression profile of a tissue sample, and each row corresponds to a circRNA. Higher expression levels are indicated by “red” and lower expression levels are indicated by “green”. (b) Volcano plot shows the up-regulated and down-regulated circRNAs in case2 vs control group. Higher expression levels are indicated by “red”, lower expression levels are indicated by “green”, and no significant difference is indicated by “black”. (c) KEGG analysis of circRNAs in case2 vs case1 group. (d) GO analysis of circRNAs in case2 vs case1group. (e) disease pathway analysis of circRNAs in case2 vs case1 group. (JPG 62 kb) [file 12943_2018_888_MOESM2_ESM.jpg]

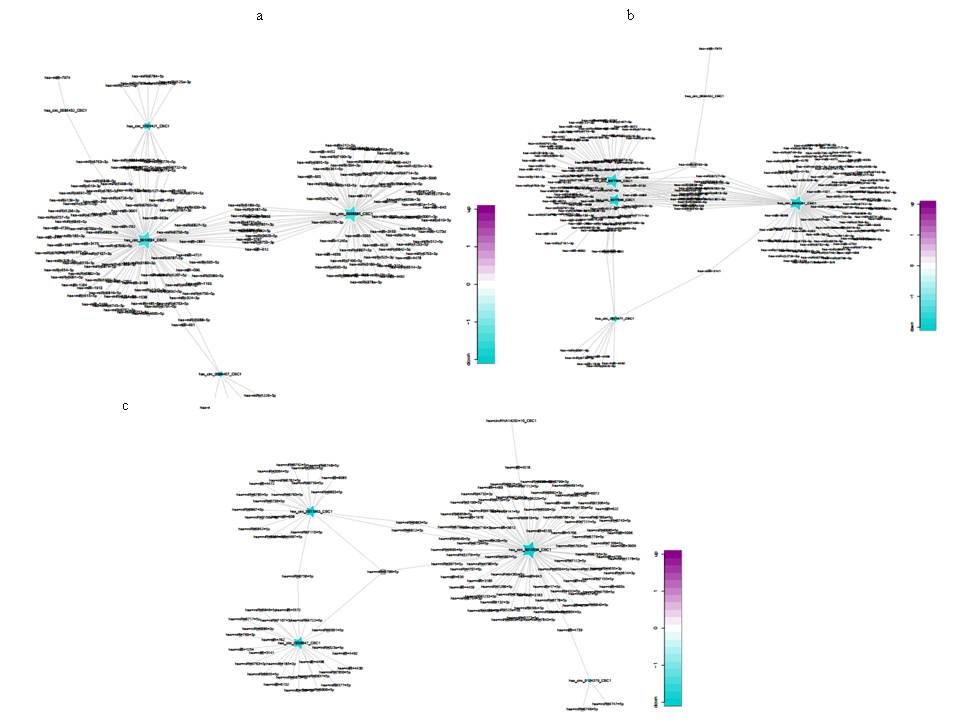

Supplement: Supplementary file 3 — Figure S3. The possible binding miRNAs of circRNAs. (a) The relationship between circRNA and possible binding miRNAs in case2 vs control group. (b) The relation of circRNA and possible binding miRNAs in case2 vs control group. (c) The relationship between circRNA and possible binding miRNAs in case2 vs case1 group. (JPG 58 kb) [file 12943_2018_888_MOESM3_ESM.jpg]

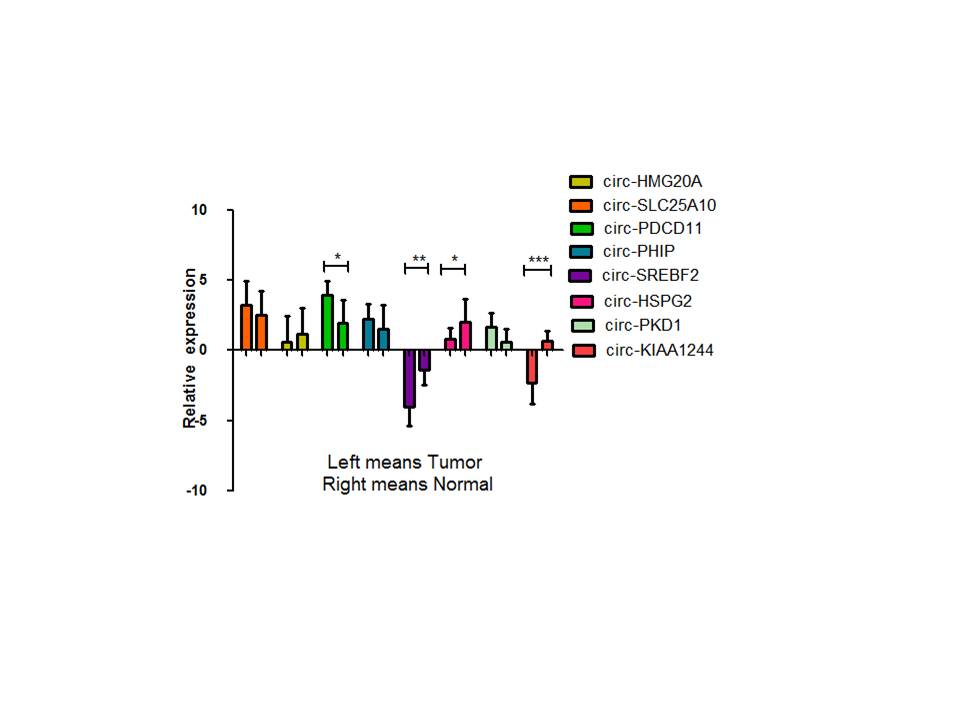

Supplement: Supplementary file 4 — Figure S4. A total of 8 circRNAs based on the multiple fold difference between the expression of GC plasmas and normal controls were verified that in a small sample of plasmas by using qRT-PCR. *P<0.05, **P<0.01,***P<0.001. (JPG 31 kb) [file 12943_2018_888_MOESM4_ESM.jpg]
