# Supplementary material for: CircRNA microarray profiling identifies a novel circulating biomarker for detection of gastric cancer
Source: Mol Cancer. 2018 Sep 20;17:137. doi: 10.1186/s12943-018-0888-8 (PMC6147053; doi:10.1186/s12943-018-0888-8)
Supplement: Supplementary file 5 — Table S1. The association of circ-KIAA1244 expression (-ΔCt) in plasma with baseline demographics of patients with GC. Table S2. Univariate and multivariate analysis for overall survival. (DOCX 24 kb) [file 12943_2018_888_MOESM5_ESM.docx]

Table S1. The association of circ-KIAA1244 expression (-ΔCt) in plasma with baseline demographics of patients with GC

| Variables | No. of patients (%) | Mean ± SD | P |
| --- | --- | --- | --- |
| Age(year) |  |  | 0.114 |
| ≥65 | 33 | -2.5366± 2.0874 |  |
| ＜65 | 29 | -3.5015± 2.6377 |  |
| Gender |  |  | 0.363 |
| Female | 15 | -2.4952±2.2609 |  |
| Male | 47 | -3.1452±2.4328 |  |
| Diameter |  |  | 0.994 |
| ≥3(cm) | 42 | -2.9897±2.3492 |  |
| ＜3(cm) | 20 | -2.9844±2.5376 |  |
| Differentiation |  |  | 0.222 |
| High | 14 | -2.2969±2.4482 |  |
| Low/Middle | 48 | -3.1895±2.3613 |  |
| Lymphatic metastasis |  |  | 0.049* |
| Positive | 33 | -3.5457±1.8953 |  |
| Negative | 29 | -2.3533±2.7498 |  |
| TNM Stage |  |  | 0.011* |
| I-II | 37 | -2.3617±2.2994 |  |
| III-IV | 25 | -3.9149±2.2571 |  |

Note: SD: Standard deviation ;**P*＜0.05.

Table S2. Univariate and multivariate analysis for overall survival

| Variable | Univariate  （P value） | Risk ratio | Multivariate  （P value） | Risk ratio |
| --- | --- | --- | --- | --- |
| Age  (≥65y vs ＜65y) | 0.857 | 1.053（0.556-1.994） | 0.247 | 1.506(0.753-3.010) |
| Gender  (Female vs Male) | 0.178 | 1.925(0.743-4.987) | 0.722 | 1.197(0.445-3.219) |
| Diameter  (≥3cm vs ＜3cm) | 0.064 | 1.885(0.964-3.686) | 0.031* | 2.264(1.078-4.756) |
| Differentiation  (High vs Low/Middle) | 0.613 | 0.819(0.377-1.778) | 0.210 | 0.590(0.259-1.346) |
| Lymphatic metastasis  (positive vs negative ) | 0.070 | 1.799(0.953-3.396) | 0.030* | 2.135(1.078-4.230) |
| TNM Stage(III-IV vs I-II) | 0.047* | 1.968(1.008-3.845) | 0.030* | 2.257(1.081-4.710) |
| circ-KIAA1244 expression  (High vs Low ) | 0.007* | 3.138(1.368-7.196) | 0.023* | 2.785(1.150-6.741) |

Note: **P*＜0.05.
